# Optimisation of Q.Clear (BSREM) Reconstruction Parameters for ^123^I and ^177^Lu Imaging with a 360° CZT Detector

**DOI:** 10.3390/bioengineering13091004

**Published:** 2026-08-28

**Authors:** Rafaela Carvalhais, Joana Teixeira, Vera Antunes, João Santos

**Affiliations:** 1 Faculty of Sciences, University of Porto, 4169-007 Porto, Portugal; 2 Medical Physics, Radiobiology and Radiation Protection Group, IPO Porto Research Center (CI-IPOP), Portuguese Institute of Oncology of Porto (IPO Porto), 4200-072 Porto, Portugal; joanamariateixeira@ipoporto.min-saude.pt (J.T.); vera.antunes@ipoporto.min-saude.pt (V.A.); joao.santos@ipoporto.min-saude.pt (J.S.); 3 School of Medicine and Biomedical Sciences (ICBAS), University of Porto, 4050-313 Porto, Portugal

**Keywords:** SPECT image reconstruction, quantitative SPECT, Q.Clear, BSREM, OSEM

## Abstract

Accurate quantitative SPECT depends strongly on the reconstruction protocol, particularly for penalised-likelihood reconstruction algorithms, such as Q.Clear (BSREM). This study aimed to optimise Q.Clear reconstruction parameters for ^123^I and ^177^Lu imaging. SPECT images of a NEMA IEC PET Body phantom filled with ^123^I and ^177^Lu were acquired on a ring-shaped CZT camera and on a conventional Anger camera. The images were reconstructed using Q.Clear and OSEM and evaluated using quantitative image-quality metrics. Q.Clear was optimised for each radionuclide. The ^123^I dataset was reconstructed using different combinations of the regularisation parameters β and γ. The ^177^Lu dataset was reconstructed using literature-reported parameters, and the best-performing reconstruction was fine-tuned. The best-performing Q.Clear reconstructions were compared with corresponding OSEM reconstructions. Overall, increasing β reduced the recovery coefficients, background variability, and contrast recovery coefficients while increasing the signal-to-noise ratio. γ had no appreciable influence on the metrics within the investigated range. The candidate optimised StarGuide/Q.Clear reconstructions outperformed the standard Symbia/OSEM reconstructions in terms of activity recovery, whereas Symbia/OSEM produced lower image noise for most of the analysed VOIs. This study identified settings associated with improved quantitative metrics within the evaluated parameter space, with potential implications for quantitative imaging requiring clinical validation. Nevertheless, multiple reconstruction parameter combinations remain unexplored.

## 1. Introduction

Single-photon emission computed tomography (SPECT) has traditionally been regarded as a non-quantitative nuclear medicine imaging modality. However, recent developments in SPECT detection technology and reconstruction software have enabled accurate quantitative SPECT, with applications in targeted radionuclide therapy (TRT), personalised dosimetry, and theranostics [1,2]. Among these developments is the increasing adoption of cadmium zinc telluride (CZT) detectors, which offer higher energy resolution, greater sensitivity, and more innovative acquisition geometries compared with their Anger counterparts [3].

The most recent innovation in SPECT detection technology is the introduction of 360° CZT whole-body SPECT systems, comprising twelve CZT detectors arranged in a ring-shaped gantry. One such system is the StarGuide SPECT/CT system (GE Healthcare, Haifa, Israel), introduced in 2021 [4], which is the focus of this study. StarGuide has been successfully implemented in clinical routine, providing image quality comparable to that of conventional Anger cameras [5], reducing whole-body scan acquisition times [6], and demonstrating higher energy and spatial resolution compared with conventional systems [7]. StarGuide has also been shown to allow accurate activity quantification in ^177^Lu imaging [8].

In addition to the well-established ordered subset expectation maximisation (OSEM) algorithm [9], the StarGuide system allows images to be reconstructed using the block sequential regularised expectation maximisation (BSREM) algorithm [10], commercially known as Q.Clear. Penalised-likelihood (PL) algorithms, such as Q.Clear, attempt to overcome one of the main limitations of OSEM—the increase in image noise with an increasing number of iterations [11]—by incorporating prior information into the reconstruction process through regularisation terms, guiding the reconstruction towards more acceptable solutions, such as smoother images [12].

Regarding StarGuide’s reconstruction capabilities, most studies have either used the OSEM algorithm [7,13,14] or adopted the manufacturer’s recommended Q.Clear settings [4,6,7]. Manufacturer-recommended settings may not always be optimised for quantitative imaging and may instead prioritise diagnostic image quality [4], highlighting the need for systematic exploration of reconstruction parameters for quantitative purposes. More recently, optimisation studies focusing mainly on varying parameters such as the number of iterations, subsets, and the regularisation parameters, and using either quantitative or qualitative analysis to determine optimal reconstruction settings, have emerged [5,8,15,16]. However, there is still no consensus regarding the optimal Q.Clear reconstruction parameters for quantitative StarGuide imaging. Additionally, quantitative accuracy continues to be heavily dependent on the reconstruction settings, particularly for PL methods. Furthermore, the reproducibility and comparability of results are compromised by the lack of standardisation across systems, especially between the new CZT-based systems and conventional Anger cameras. In this context, the present study systematically evaluates the Q.Clear reconstruction parameters for quantitative ^123^I and ^177^Lu StarGuide imaging using NEMA phantom data and a range of quantitative and image-quality metrics. This approach enables a cross-radionuclide assessment of reconstruction-parameter behaviour and provides a quantitative framework for the evaluation of Q.Clear settings.

The radionuclides used in theranostics that have driven the need for accurate quantitative SPECT include ^123^I and ^177^Lu. ^123^I emits γ photons with an energy of 159 keV (83%) [17]. It is commonly paired with ^131^*I* ($\beta^{-}$ emitter) for treatment, pre- and post-therapeutic assessment of differentiated thyroid cancer, and to identify and treat neuroendocrine tumours that express the norepinephrine transporter ([^123^I]-MIBG)/[^131^*I*]-MIBG) [18]. In addition to the primary photons, ^123^I emits a series of high-energy photons that contribute to septal penetration and scatter, thereby compromising image quality [19,20]. While diagnostically and dosimetrically relevant, these phenomena make its imaging challenging and further highlight the need for careful validation of reconstruction and correction strategies.

^177^Lu decays into the stable radionuclide ^177^*Hf* by $\beta^{-}$ emission. During the decay process, γ photons with energies of 113 keV (6%) and 208 keV (10%) are also emitted [21]. This radionuclide has been increasing in popularity due to its use in the treatment of metastatic castration-resistant prostate cancer ([^177^Lu]Lu-PSMA-617) and inoperable gastroenteropancreatic neuroendocrine tumours ([^177^Lu]Lu-DOTATATE) [18]. Its central role in molecular radiotherapy, theranostics, and patient-specific dosimetry makes it a natural and clinically important target for quantitative CZT-SPECT optimisation.

The main objective of this study was to explore, assess, and optimise the reconstruction settings of the Q.Clear reconstruction algorithm for ^123^I and ^177^Lu imaging, with a particular focus on the algorithm’s regularisation parameters and quantitative metric analysis. This study successfully characterised the behaviour of the Q.Clear regularisation parameters, and, within the parameter range studied, identified candidate optimised reconstruction protocols, forming the foundation for future optimisation studies.

## 2. Materials and Methods

### 2.1. StarGuide SPECT/CT

The StarGuide SPECT/CT system combines GE Healthcare’s Optima CT540 CT scanner (GE Healthcare, Haifa, Israel) with a ring-shaped SPECT gantry, in which 12 CZT detectors are evenly distributed. Each detector is composed of 7 CZT modules, each containing a 16×16 pixel matrix with an individual pixel size of $2.46\times 2.46\;{\mathrm{mm}}^{2}$ [8]. Each crystal is 7.25mm thick, granting StarGuide an imaging energy range of 40–279 keV [22]. Additionally, each detector is coupled to a fixed low-energy high-resolution (LEHR) tungsten collimator, enabling the imaging of low- and medium-energy radionuclides without the need for collimator exchange and allowing the acquisition of both ^177^Lu 113 keV and 208 keV photons [8,13,22].

The detector arms can move independently in the radial direction, either inward or outward. They can also rotate simultaneously with the gantry, enabling acquisitions at various angular positions. For a given angular position, the tip of each detector arm swivels during the scan [4,7,8]. A sensor ring, comprising transmitting and receiving infrared diodes, is coupled to the front of the SPECT gantry. Using this infrared technology, an optical scout of the imaged object is performed prior to the SPECT acquisition. The data acquired during this scout are used to define the table position and optimise the radial position of each detector arm [8,22].

### 2.2. Q.Clear

Q.Clear allows reconstruction using relative difference prior (RDP) [23] or median root prior (MRP) [24] regularisation. In this study, only Q.Clear with RDP regularisation was used. In this case, Q.Clear’s objective function, that is, the function to be maximised during the reconstruction process, can be written as

(1) $$\phi \left(f\right)=\sum_{i}g_{i}\;\log\left({\left[Pf\right]}_{i}+r_{i}\right)-\left({\left[Pf\right]}_{i}+r_{i}\right)-\beta R\left(f\right),$$

with

(2) $$R\left(f\right)=\sum_{j}\sum_{k\in N_{j}}w_{jk}\frac{{\left(f_{j}-f_{k}\right)}^{2}}{\left(f_{j}+f_{k}\right)+\gamma \;\left|f_{j}-f_{k}\right|}\;.$$

In Equation (1), *i* denotes the measurement index; $g_{i}$ represents the acquired projection data for measurement *i*; f is a vector representing the image activity; $r_{i}$ represents scatter and background events for measurement *i*; *P* is the system matrix; and β corresponds to the penalty parameter, controlling the strength of the regularisation used to reduce image noise [25,26,27]. In Equation (2), *j* denotes the voxel index, *k* denotes the index of a voxel neighbouring voxel *j*, $N_{j}$ represents the neighbourhood of voxel *j*, $w_{jk}$ is a weighting factor related to the distance between voxels *j* and *k*, and γ is a parameter that controls the shape of the RDP prior. In particular, γ controls the degree of edge preservation, with higher values producing sharper images [23,25,28].

In addition to the regularisation parameters γ and β, other reconstruction settings can also be defined in the StarGuide system, including the number of iterations, the number of subsets, the application of CT-based attenuation correction and energy window-based scatter correction, the use of tail photons in the reconstruction process to account for the characteristic CZT tailing effects, the voxel size, and the application of post-reconstruction filters. One such filter is Clarity 3D (C3D), described by GE Healthcare as an edge-preserving filter, for which the filter strength can be selected, with optional contrast enhancement [22]. Furthermore, the penalty strength β can be weighted according to a sensitivity map derived from the CT attenuation map by enabling the By Sens tool [8].

### 2.3. Phantom

This study used a NEMA IEC PET Body phantom (Data Spectrum, Durham, NC, USA) equipped with a lung insert (260mL) and an insert containing six fillable hollow spheres (internal diameters: 10, 13, 17, 22, 28, and 37mm; volumes: 0.5, 1.2, 2.6, 5.6, 11.5, and 26.5mL), hereafter referred to as the NEMA phantom.

On separate occasions, the spheres and background of the NEMA phantom were filled with 0.068MBq/mL and 0.007MBq/mL of ^123^I, and 0.730MBq/mL and 0.079MBq/mL of ^177^Lu, respectively, corresponding to sphere-to-background ratios of 10 and 9. The phantom was subsequently acquired on the StarGuide SPECT/CT system and on a conventional dual-head Anger camera, the Symbia Pro.specta SPECT/CT system (Siemens Healthineers, Erlangen, Germany), for 15 min.

### 2.4. Image Acquisition

The acquisition parameters used for both systems are presented in Table 1. For the CT and SPECT scans, the acquisition settings were those routinely used in standard clinical practice at our institution for each system.

For StarGuide, projections of the NEMA phantom were acquired using the continuous sweep mode over 15 min. In this mode, the detector tips swivel during acquisition, acquiring data in 2° steps. The system automatically defines six gantry positions for the detector assembly to assume. At times scheduled by the system according to the total acquisition time, the detector arms stop acquiring, recoil, and rotate 5° clockwise before resuming acquisition in the new gantry position. For Symbia Pro.specta acquisitions, each detector performed a 180° body-contour trajectory. At each acquisition angle, each detector acquired 60 views over 15s.

For ^177^Lu, two 15-min acquisitions of the NEMA phantom were ultimately available due to technical issues unrelated to this study. Following the appropriate decay corrections, both datasets were included in the optimisation procedure. The only difference between the acquisitions was the size of the reconstructed SPECT image, as shown in Table 1.

### 2.5. Image Reconstruction

The StarGuide images were reconstructed on SmartConsole (version 1.1), StarGuide’s web-based reconstruction platform, using Q.Clear with RDP regularisation, while the Symbia Pro.specta images were reconstructed using OSEM. For StarGuide, Q.Clear’s reconstruction parameters were optimised following the strategies reported below. For Symbia Pro.specta, the clinical reconstruction protocols used at our institution were considered.

#### 2.5.1. ^123^I

To study the influence of Q.Clear’s regularisation parameters β and γ on the image-quality and quantitative metrics, twenty images were reconstructed, considering all possible combinations of the following values: β=0.01,0.05,0.1,0.5,1 and γ=0.1,0.5,1,3. Following the clinical reconstruction protocol considered at our institution, the number of iterations was fixed at 40 and the number of subsets was fixed at 10. Additionally, the tail photons were integrated into the reconstruction process, the By Sens tool was enabled, and Clarity 3D was used with a power of 0.01 and no contrast enhancement. A number was attributed to each reconstruction protocol to facilitate their identification (Table 2).

For Symbia Pro.specta, the following reconstruction parameters were considered: 18 iterations, 4 subsets, and a post-reconstruction Gaussian filter (FWHM: 10mm). All reconstructions, regardless of the system, used CT-based attenuation correction and scatter correction, with this last one according to the windows defined in Table 1.

#### 2.5.2. ^177^Lu

When reconstructing ^177^Lu on StarGuide, the reconstruction settings for both the 113keV and 208keV energy windows must be defined. The system then reconstructs both images, and a third image, corresponding to the voxel-wise average of the first two [4], can also be reconstructed. All the Q.Clear protocols tested are presented in Table 3. The data from the first ^177^Lu acquisition were reconstructed using protocols ^177^*Lu*_*R*1 to ^177^*Lu*_*R*4, whereas the remaining reconstruction protocols were applied to the data from the second acquisition.

To optimise Q.Clear’s reconstruction parameters for this radionuclide, the following strategy was followed: first, three literature-reported settings (protocols ^177^*Lu*_*R*1 to protocols ^177^*Lu*_*R*3) and one clinical reconstruction protocol used at our institution were considered (^177^*Lu*_*R*4). The three literature-reported settings correspond to the optimised Q.Clear with RDP regularisation established by Danieli et al. [8] in their quantitative ^177^Lu study (protocol ^177^*Lu*_*R*1); the factory reconstruction protocol used in the same study (protocol ^177^*Lu*_*R*2); and the settings used by Hoog et al. [4] in their comparative study of whole-body CZT scanners (protocol ^177^*Lu*_*R*3).

Based on a quantitative analysis of these protocols, a new protocol, protocol ^177^*Lu*_*R*5, was created by combining the following reconstruction settings from protocols ^177^*Lu*_*R*2 and ^177^*Lu*_*R*4:

- Protocol ^177^*Lu*_*R*5’s 113keV energy window: protocol ^177^*Lu*_*R*2’s 113keV energy window reconstruction settings.
- Protocol ^177^*Lu*_*R*5’s 208keV energy window: protocol ^177^*Lu*_*R*4’s 208keV energy window reconstruction settings.

These settings were selected based on the complementary activity recovery performance observed for each energy window, as further detailed in Section 3.2.1.

This protocol was then fine-tuned with the aim of reducing image noise. Following the quantitative analysis being conducted, alterations were made to the 113keV window, considering two higher β values (0.2 and 0.3; protocols ^177^*Lu*_*R*6 and ^177^*Lu*_*R*7) and a reduction in the number of iterations (^177^*Lu*_*R*8). To match the 208keV window, the Clarity 3D filter was enabled for these protocols, with a power of 0.01. To see the influence of this setting, protocol ^177^*Lu*_*R*5 was repeated but with Clarity 3D applied to the 113keV, originating protocol ^177^*Lu*_*R*9.

The optimisation procedure described above and further detailed in Section 2.6 focused primarily on the averaged image reconstructed by the StarGuide system. The two energy peaks provide complementary characteristics that may contribute to the overall quantitative and qualitative performance of the reconstructed image. In particular, the 208keV emission has a higher abundance than the 113keV emission, providing higher count statistics and potentially reducing image noise. Conversely, the 113keV emission is less susceptible to septal penetration, which may contribute to improved spatial resolution and sharper object boundaries. Therefore, voxel-wise averaging of the two energy-window images was used to combine these complementary characteristics in a single reconstructed image.

For Symbia Pro.specta, the following reconstruction parameters were considered: 12 iterations, 8 subsets, and a post-reconstruction Gaussian filter (FWHM: 4mm). Once again, all reconstructions, regardless of the system, used CT-based attenuation correction and scatter correction.

### 2.6. Image Analysis

Image analysis was performed using MIM Software (MIM Software Inc., Cleveland, OH, USA, version 7.3.4) [29] and Python (version 3.14). To systematically assess the NEMA phantom images, the dedicated NEMA phantom analysis workflow available in MIM was modified to enable automatic conversion from counts to activity concentration, including automatic determination of the calibration factor. A detailed description of the developed workflow is provided in Section S1 of the Supplementary Material.

For the calculation of image-quality metrics, six VOIs corresponding to the spherical inserts of the NEMA phantom were defined, together with six identical VOIs placed in the phantom background (hereafter referred to as clone spheres) to estimate the background contribution, allowing subtraction of the background activity from the measured activity within each NEMA sphere, thereby isolating the signal truly attributable to the phantom’s spheres from that arising from the phantom’s background.

Recovery coefficients (RCs) were determined using the total activity within each NEMA sphere, corrected for background contributions using the corresponding clone sphere ($A_{measured}=A_{NEMA}-A_{Clone}$), and the expected activity within each VOI, $A_{true}$, obtained by multiplying the decay-corrected activity concentration at the acquisition time by the VOI volume:

(3) $$RC=\frac{A_{measured}}{A_{true}}\;.$$

The RCs of the candidate optimised Q.Clear protocols and the OSEM reconstructions were fitted to Equation (4) using the model proposed by Gear et al. [30] (hereafter referred to as the EANM model), consisting of a two-parameter ($b_{1}$ and $b_{2}$) logistic function:

(4) $$RC\left(v\right)=1-\frac{1}{1+{\left(\frac{v}{b_{1}}\right)}^{b_{2}}}\;.$$

The goodness of fit was assessed using the coefficient of determination ($R^{2}$).

Image noise was quantified using the percentage background variability (BV), defined as

(5) $$BV=\frac{\sqrt{\frac{1}{K-1}\sum_{k=1}^{K}{\left(c_{k}-\bar{c}\right)}^{2}}}{\bar{c}}\times 100\%\;,$$

where $c_{k}$ is the activity concentration within VOI *k*, and $\bar{c}$ is the mean activity concentration across the *K* VOIs [31]. The BV was computed for five sets of 25 spherical background VOIs with volumes of 0.5, 2.6, 26.5, 50, and 100mL.

The signal-to-noise ratio (SNR) was computed for each NEMA sphere according to [32]:

(6) $$SNR=\frac{{\bar{c}}_{sphere}-{\bar{c}}_{clone}}{SD_{clone}}\;,$$

where ${\bar{c}}_{sphere}$ is the mean activity concentration within the NEMA sphere, ${\bar{c}}_{clone}$ is the mean activity concentration within the corresponding clone sphere, and $SD_{clone}$ is the standard deviation of the activity concentration within the clone sphere.

The percentage contrast recovery coefficient (CRC) was also determined for each NEMA sphere, using

(7) $$CRC=\frac{\frac{{\bar{c}}_{sphere}}{{\bar{c}}_{clone}}-1}{\frac{a_{sphere}}{a_{clone}}-1}\;\times 100\%\;,$$

where $a_{sphere}$ and $a_{clone}$ are the true activity concentrations used to fill the NEMA spheres and the background, respectively [33].

## 3. Results

### 3.1. *^123^*I

#### 3.1.1. Optimisation of Q.Clear Reconstruction Parameters

**Figure 1:**
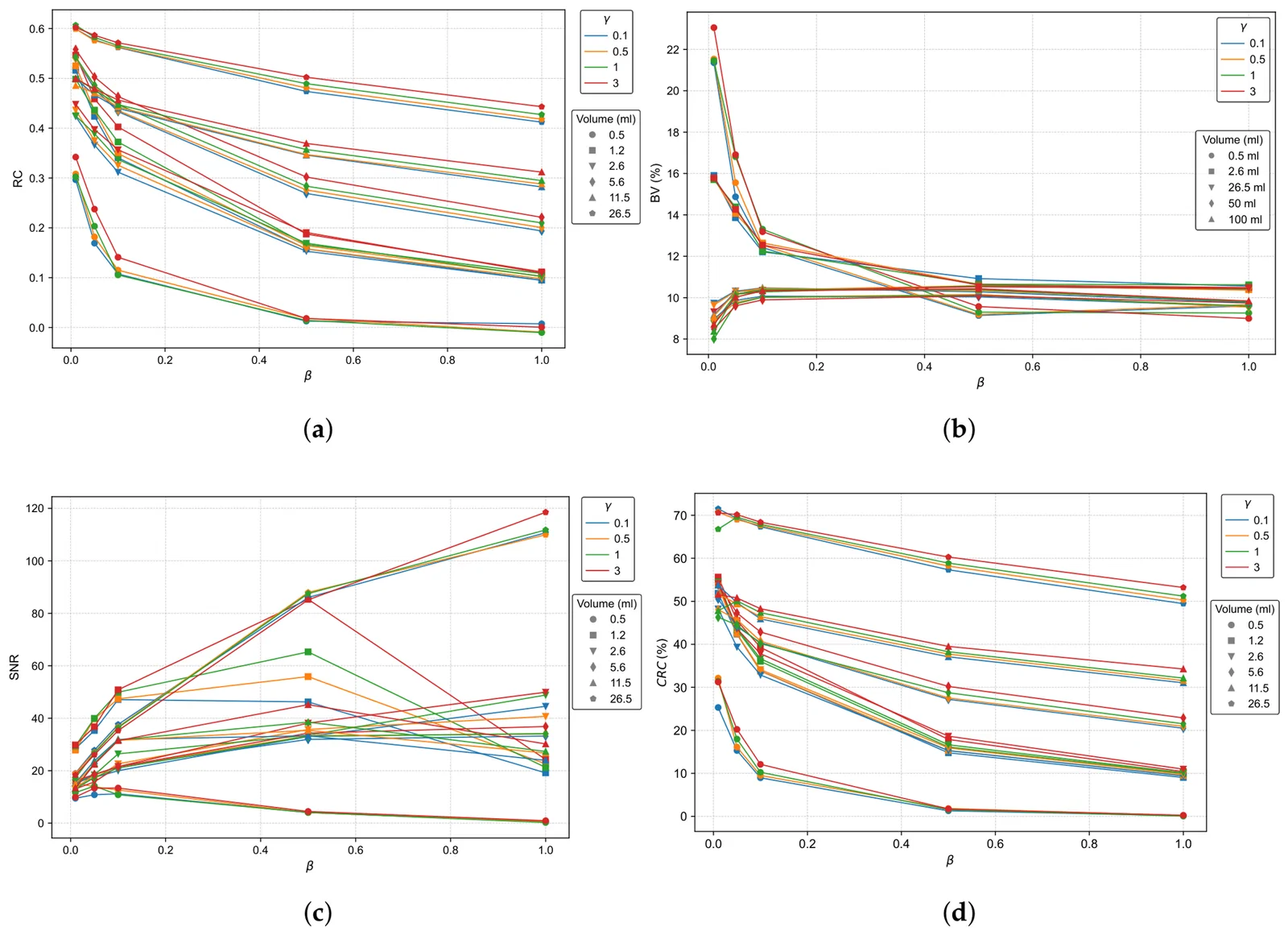
Results of the systematic evaluation of β: (**a**) Recovery coefficients as a function of β. (**b**) Background variability as a function of β. (**c**) Signal-to-noise ratio as a function of β. (**d**) Contrast recovery coefficients as a function of β.

The RC, BV, SNR, and CRC values obtained for each ^123^I Q.Clear protocol are presented in Figure 1. A clear trade-off between activity and contrast recovery and image noise is observed as β varies. Overall, increasing β reduces the RCs and CRCs while generally increasing the SNR and, for the smaller VOI sets, decreasing the BV.

The suggested β and γ ranges were selected based on the observed behaviour across all the evaluated metrics, with particular emphasis on maintaining detectability of the 0.5mL sphere while limiting image noise. Specifically, β≥0.5 was excluded because it consistently produced the lowest RCs and CRCs across all sphere volumes and the lowest SNR for the 0.5mL sphere. In addition, β=0.01 was excluded because it resulted in the highest BV for the smaller spheres. Consequently, the suggested β range was defined as 0.05–0.1.

A limited influence of γ on the evaluated metrics was observed, with all metrics remaining relatively constant despite changes in γ (Supplementary Figure S2). Nevertheless, for a fixed β, the lowest RCs were generally obtained for γ=0.1, whereas the highest were obtained for γ=3, leading to the exclusion of γ=0.1. Furthermore, the highest BV values for the smallest sphere were observed for γ=3, while the lowest overall BV was obtained for γ=1. Therefore, γ=1 was selected as the upper limit of the suggested γ range, which was consequently defined as 0.5–1.

Considering the combinations of the β and γ ranges associated with the most favourable results, particularly regarding the detectability of the smallest sphere, protocols ^123^*I*_*R*7, ^123^*I*_*R*8, ^123^*I*_*R*12, and ^123^*I*_*R*13 (Table 2) were further compared with the OSEM reconstruction for all the studied metrics.

#### 3.1.2. StarGuide/Q.Clear Versus Symbia/OSEM

**Figure 2:**
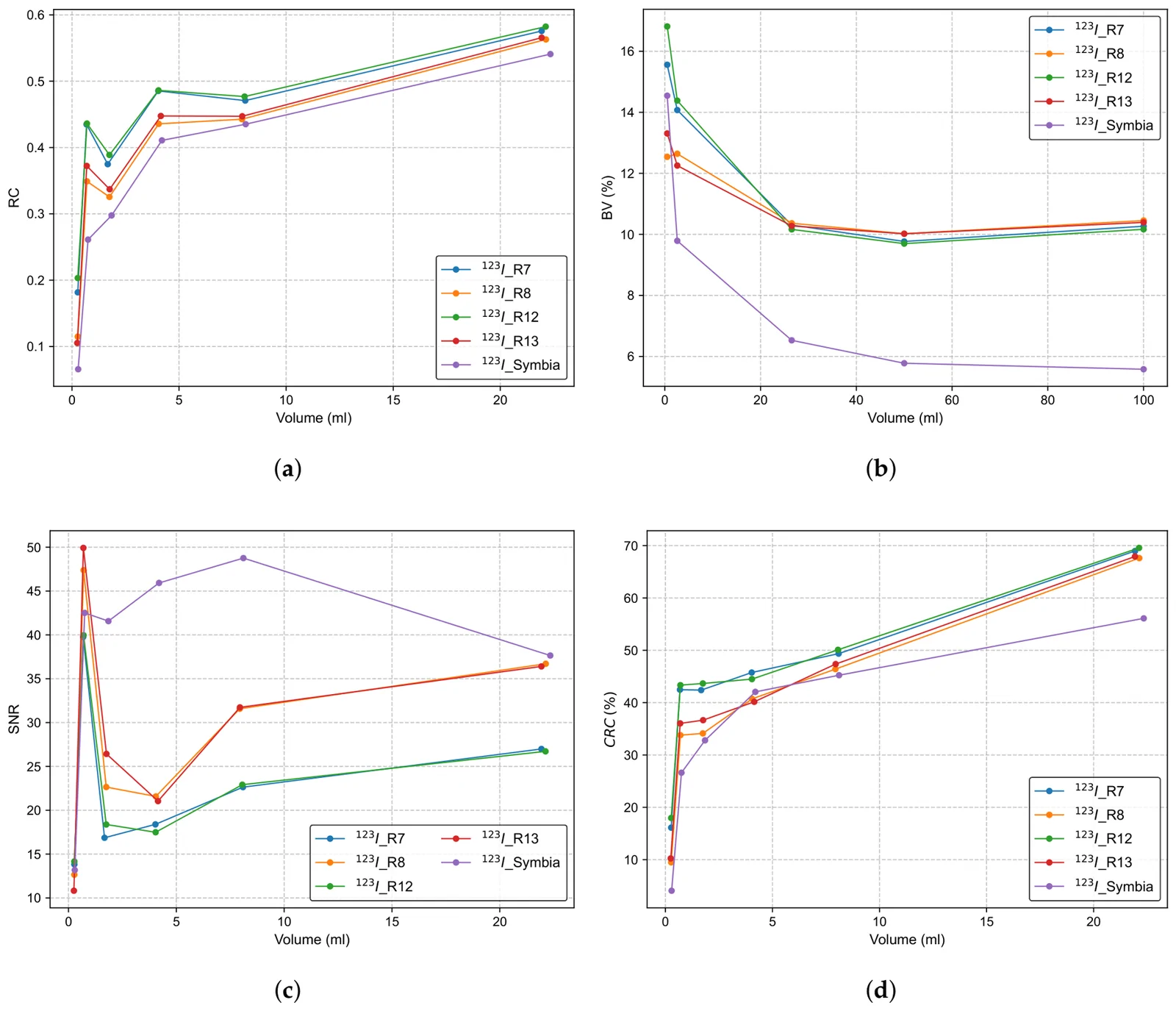
Comparison of the quantitative image-quality metrics obtained for the candidate optimised ^123^I StarGuide/Q.Clear reconstructions and for the Symbia/OSEM reconstructions: (**a**) Recovery coefficients. (**b**) Background variability. (**c**) Signal-to-noise ratio. (**d**) Contrast recovery coefficients.

The RCs, BV, SNR, and CRCs obtained for the selected ^123^I Q.Clear protocols are compared with those obtained using the Symbia Pro.specta OSEM reconstruction (protocol ^123^*I*_*Symbia*) in Figure 2.

The StarGuide/Q.Clear reconstructions consistently presented higher RCs and, generally, CRCs than the Symbia/OSEM reconstruction. The Symbia/OSEM reconstruction globally outperformed the StarGuide/Q.Clear reconstructions in terms of noise, presenting lower BV values and higher SNR values for the majority of the analysed VOIs.

Within the Q.Clear protocols, variations in β led to the most pronounced differences observed between reconstructions, with the β=0.1 protocols presenting a lower BV and higher SNR, at the expense of reduced RCs and CRCs due to stronger regularisation.

Figure 2a demonstrates a clear reduction in the RCs in the transition from the 1.2mL sphere to the 2.6mL sphere for all Q.Clear reconstructions. This was unexpected, as RC values typically increase with sphere volume due to the mitigation of partial volume effects that occurs as the volume increases. Additionally, all Q.Clear protocols showed an unexpectedly high peak in the SNR for the 1.2mL sphere.

**Figure 3:**
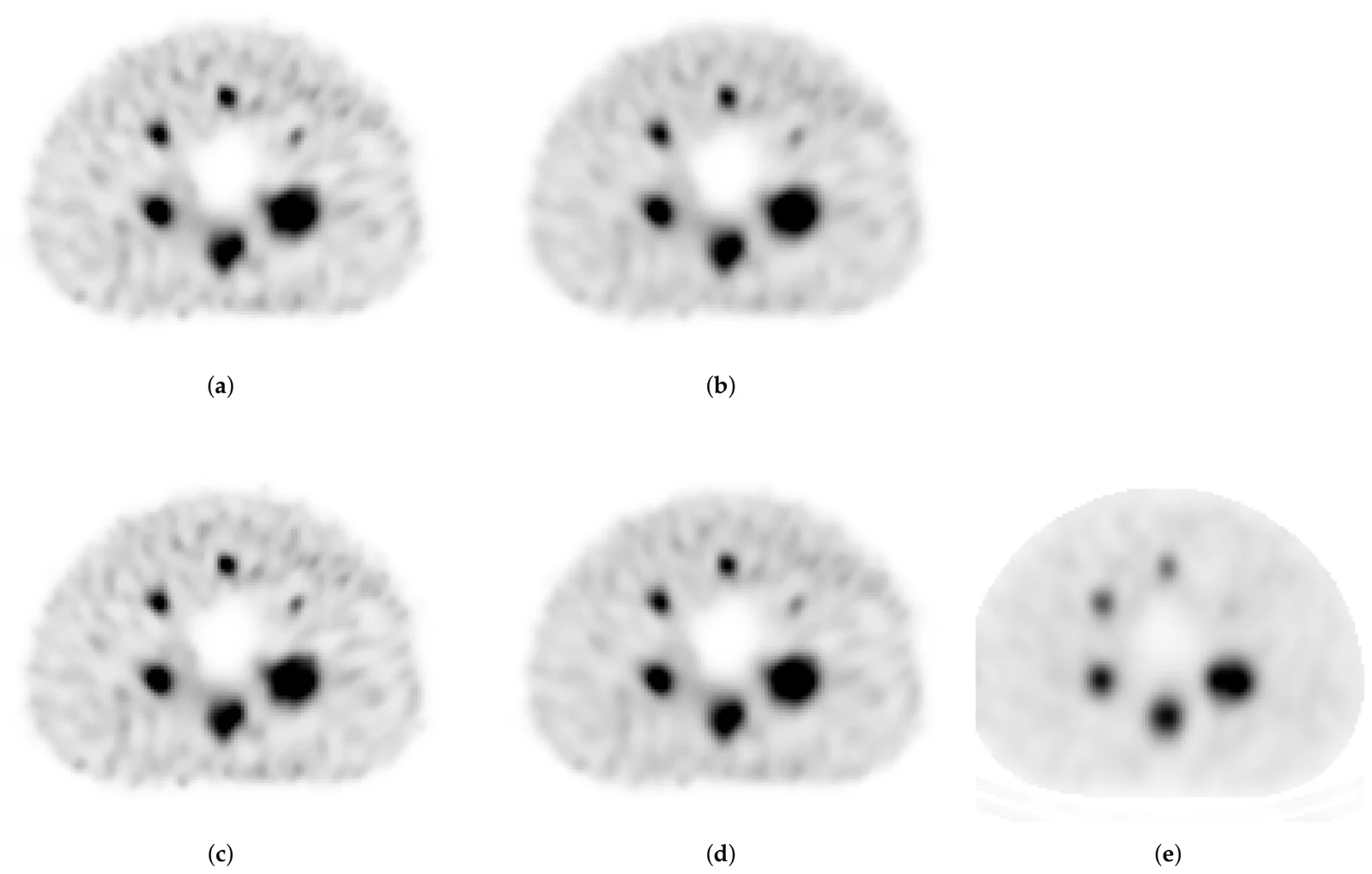
Axial views of the ^123^I-filled NEMA phantom reconstructed using the candidate optimised StarGuide/Q.Clear settings and Symbia/OSEM: (**a**) Protocol ^123^*I*_*R*7. (**b**) Protocol ^123^*I*_*R*8. (**c**) Protocol ^123^*I*_*R*12. (**d**) Protocol ^123^*I*_*R*13. (**e**) Protocol ^123^*I*_*Symbia*.

Figure 3 presents axial views of the NEMA phantom for the candidate optimised StarGuide/Q.Clear protocols (panels (a) to (d)) and the Symbia/OSEM reconstruction (panel (e)). The axial views of the NEMA phantom for all the Q.Clear protocols can be found in Supplementary Figure S3.

The 0.5mL sphere is identifiable in all the StarGuide/Q.Clear protocols, whereas it is barely visible in the Symbia/OSEM reconstruction. The phantom background is smoother and more uniform in the Symbia/OSEM reconstruction, with the StarGuide/Q.Clear reconstructions presenting a noisier and grainier background.

**Figure 4:**
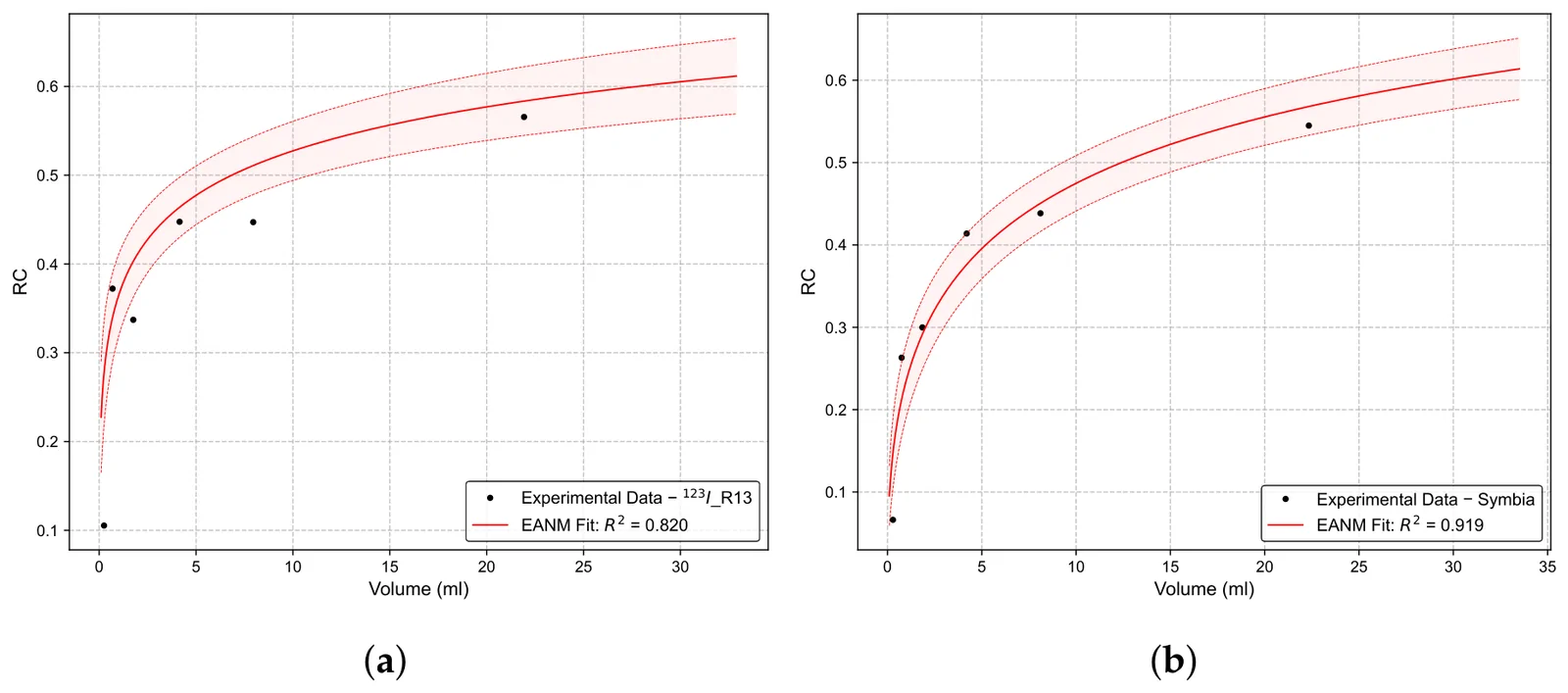
Recovery coefficients and the corresponding EANM fits for ^123^I: (**a**) ^123^*I*_*R*13 dataset. (**b**) Symbia/OSEM dataset. The dotted red lines represent the lower and upper limits of the 95% confidence band for the fitted function (red solid line).

Reconstruction protocol ^123^*I*_*R*13 was selected for further comparison with the Symbia reconstruction by fitting the RCs to the model described above (Figure 4), as it presented, in general, a higher SNR than the other Q.Clear reconstructions while maintaining, overall, higher RCs and CRCs than the Symbia/OSEM reconstruction.

The Symbia/OSEM reconstruction showed a better fit to the considered model ($R^{2}=0.919$) than the StarGuide/Q.Clear reconstruction ($R^{2}=0.820$), primarily due to the unexpected oscillatory behaviour exhibited by the StarGuide/Q.Clear RC data.

### 3.2. *^177^*Lu

#### 3.2.1. Optimisation of Q.Clear Reconstruction Parameters

An initial screening of the candidate reconstruction protocols showed that the ^177^*Lu*_*R*2 settings provided the best recovery for the 113keV image, whereas the ^177^*Lu*_*R*4 settings performed best for the 208keV image, particularly for the largest spheres (Supplementary Figures S4 and S5).

**Figure 5:**
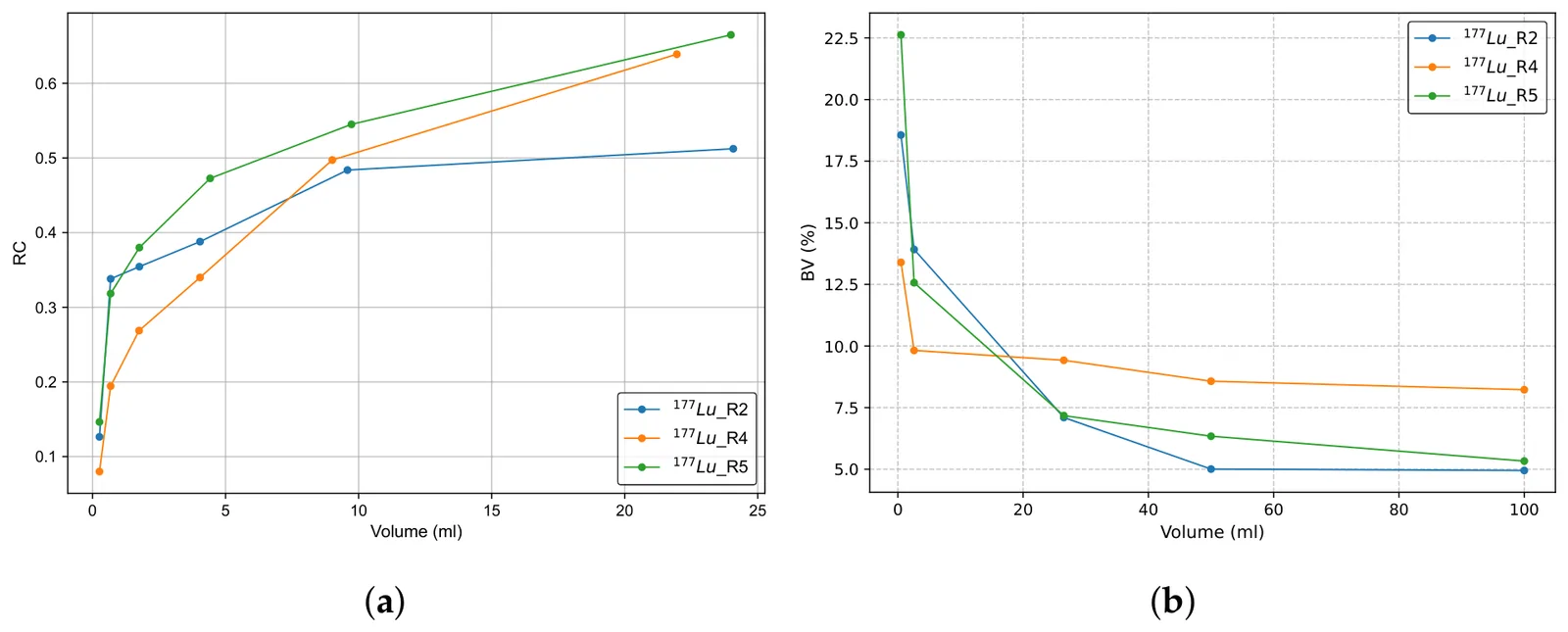

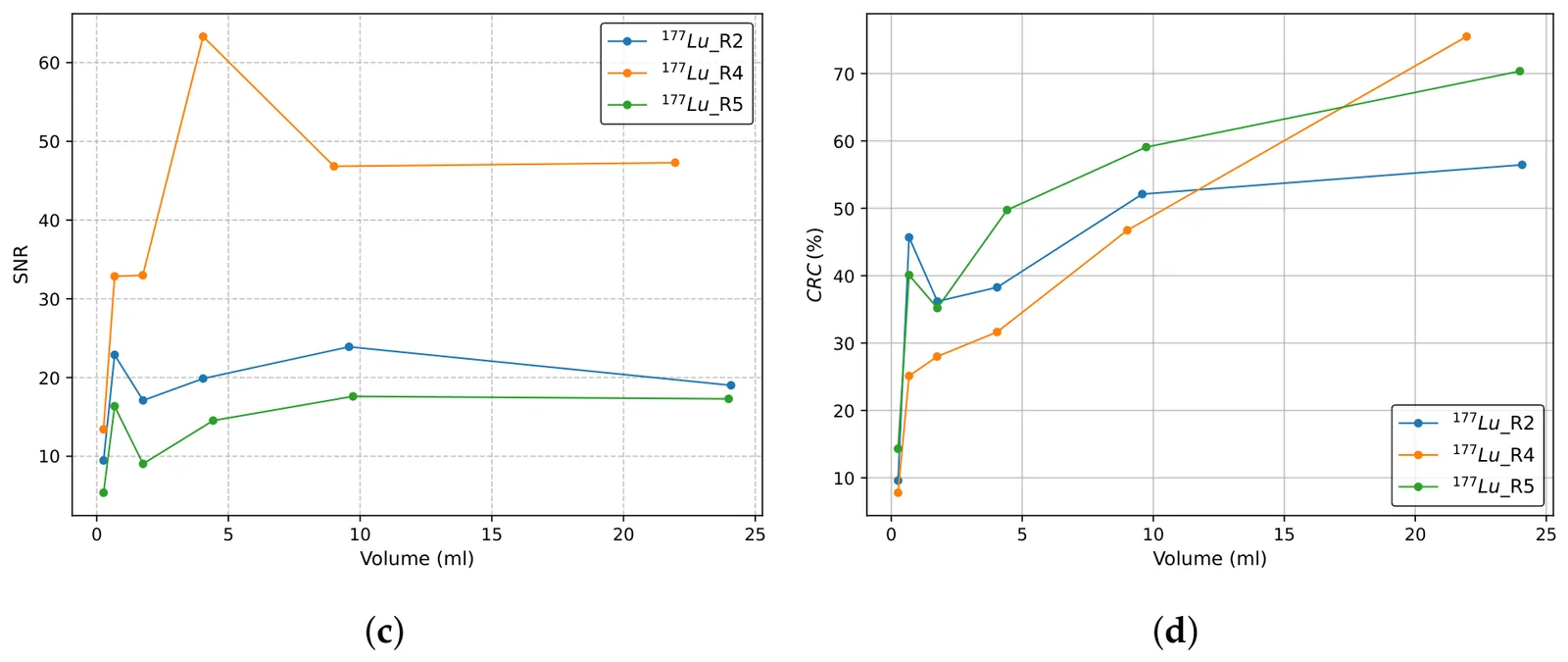
Comparison of the quantitative image-quality metrics obtained for the basis reconstruction protocols ^177^*Lu*_*R*2 and ^177^*Lu*_*R*4 and for the combined reconstruction protocol ^177^*Lu*_*R*5 using the averaged image: (**a**) Recovery coefficients. (**b**) Background variability. (**c**) Signal-to-noise ratio. (**d**) Contrast recovery coefficients.

These settings were therefore combined into protocol ^177^*Lu*_*R*5, which was compared with the two original protocols across all quantitative metrics (Figure 5).

**Figure 6:**
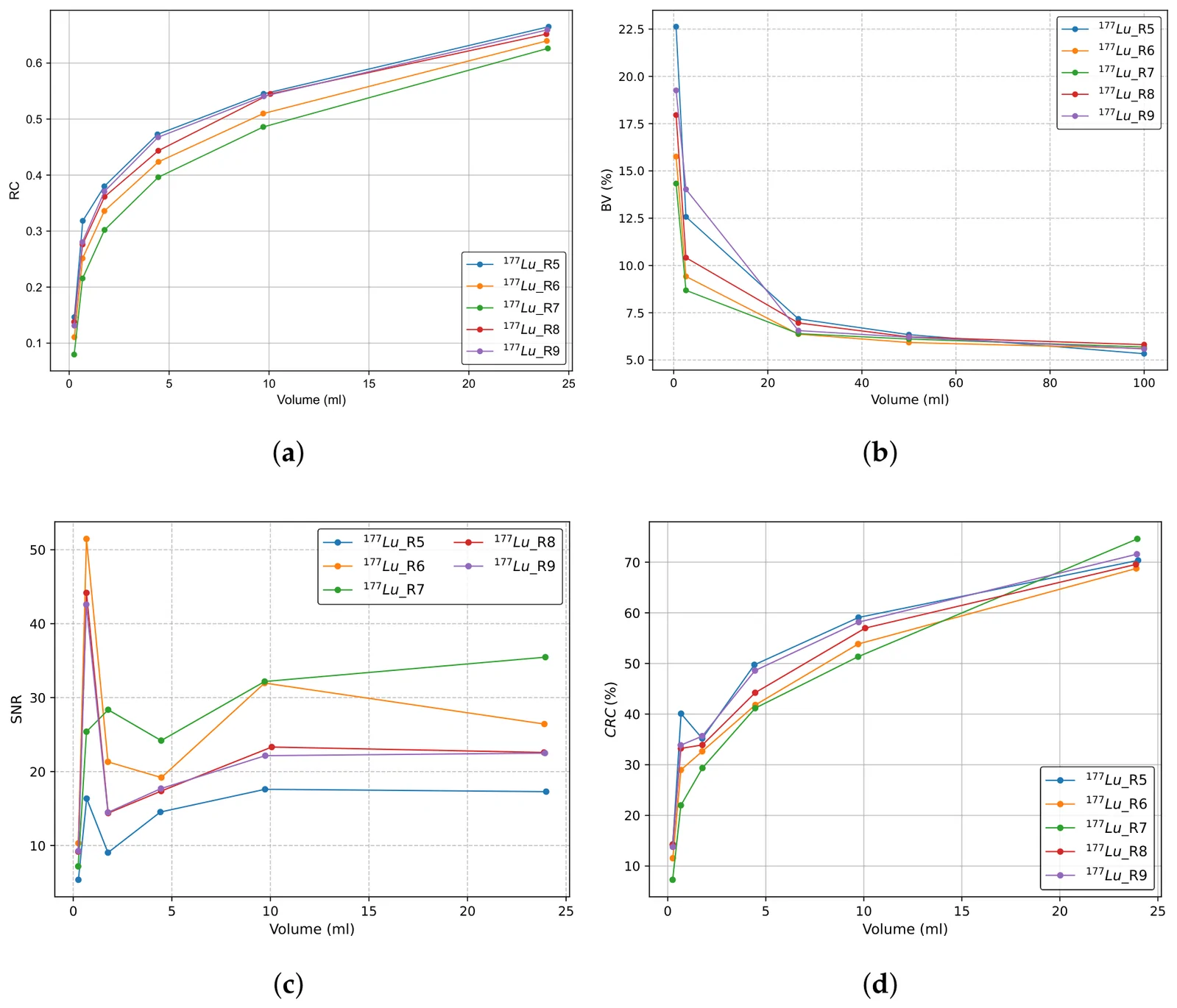
Comparison of the quantitative image-quality metrics obtained for protocols ^177^*Lu*_*R*5 to ^177^*Lu*_*R*9: (**a**) Recovery coefficients. (**b**) Background variability. (**c**) Signal-to-noise ratio. (**d**) Contrast recovery coefficients.

Protocol ^177^*Lu*_*R*5 improved the RCs and generally increased the CRCs relative to the baseline protocols, while maintaining a BV similar to protocol ^177^*Lu*_*R*2. However, this improvement was accompanied by a substantial reduction in the SNR, motivating the subsequent refinement of the 113keV reconstruction settings in protocols ^177^*Lu*_*R*6 to ^177^*Lu*_*R*9 (Figure 6).

As observed for ^123^I, increasing β improved the SNR and BV at the expense of lowering the RCs and CRCs. Reducing the number of iterations led to a higher SNR and lower image noise at the expense of lower RCs and CRCs. Enabling Clarity 3D in the 113 keV energy window had no appreciable impact on the RCs, BV, and CRCs, but did increase the SNR relative to the original protocol.

While both ^177^*Lu*_*R*6 and ^177^*Lu*_*R*7 provided overall greater improvements in the SNR when compared to the other protocols, ^177^*Lu*_*R*6 was chosen as the candidate optimised protocol within the investigated conditions as it led to the least reduction in recovery between the two. The quantitative results for the 113keV, 208keV, and averaged images for all the considered metrics for the selected protocol, as well as the axial views of the NEMA phantom for these settings, can be found in Figures S6 and S7 of the Supplementary Material, respectively.

For protocols ^177^*Lu*_*R*6, ^177^*Lu*_*R*8, and ^177^*Lu*_*R*9, a pronounced increase in the SNR was observed for the 1.2mL sphere, similar to that previously observed for the StarGuide protocols for ^123^I (Figure 2c).

#### 3.2.2. StarGuide/Q.Clear Versus Symbia/OSEM

**Figure 7:**
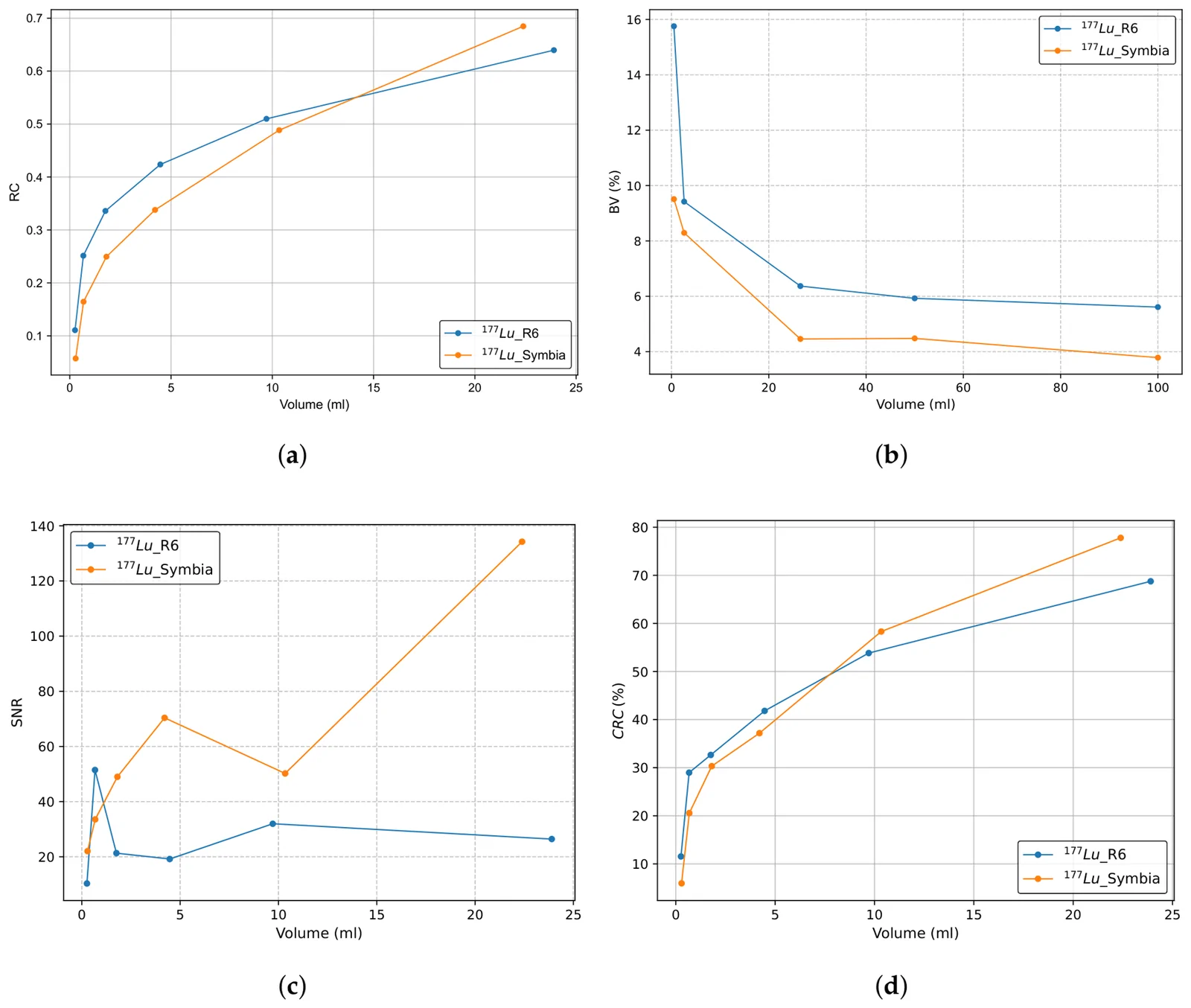
Comparison of the quantitative image-quality metrics obtained for the selected candidate optimised ^177^Lu StarGuide/Q.Clear reconstruction settings within the investigated conditions and for the Symbia/OSEM reconstruction: (**a**) Recovery coefficients. (**b**) Background variability. (**c**) Signal-to-noise ratio. (**d**) Contrast recovery coefficients.

In Figure 7, the RC, BV, SNR, and CRC values are compared between the selected candidate optimised StarGuide/Q.Clear reconstruction protocol within the studied parameter space and the Symbia/OSEM reconstruction (^177^*Lu*_*Symbia*). The RC and CRC values increase with sphere volume, as expected, with the BV decreasing accordingly.

The selected StarGuide/Q.Clear protocol globally outperformed Symbia/OSEM in terms of the RCs and CRCs, registering substantial improvements (57% and 94%, respectively) for the 0.5mL sphere compared with Symbia/OSEM. In general, and in agreement with what was observed for ^123^I, Symbia/OSEM outperformed StarGuide/Q.Clear in the noise-related metrics, achieving the highest SNR and the lowest BV.

**Figure 8:**
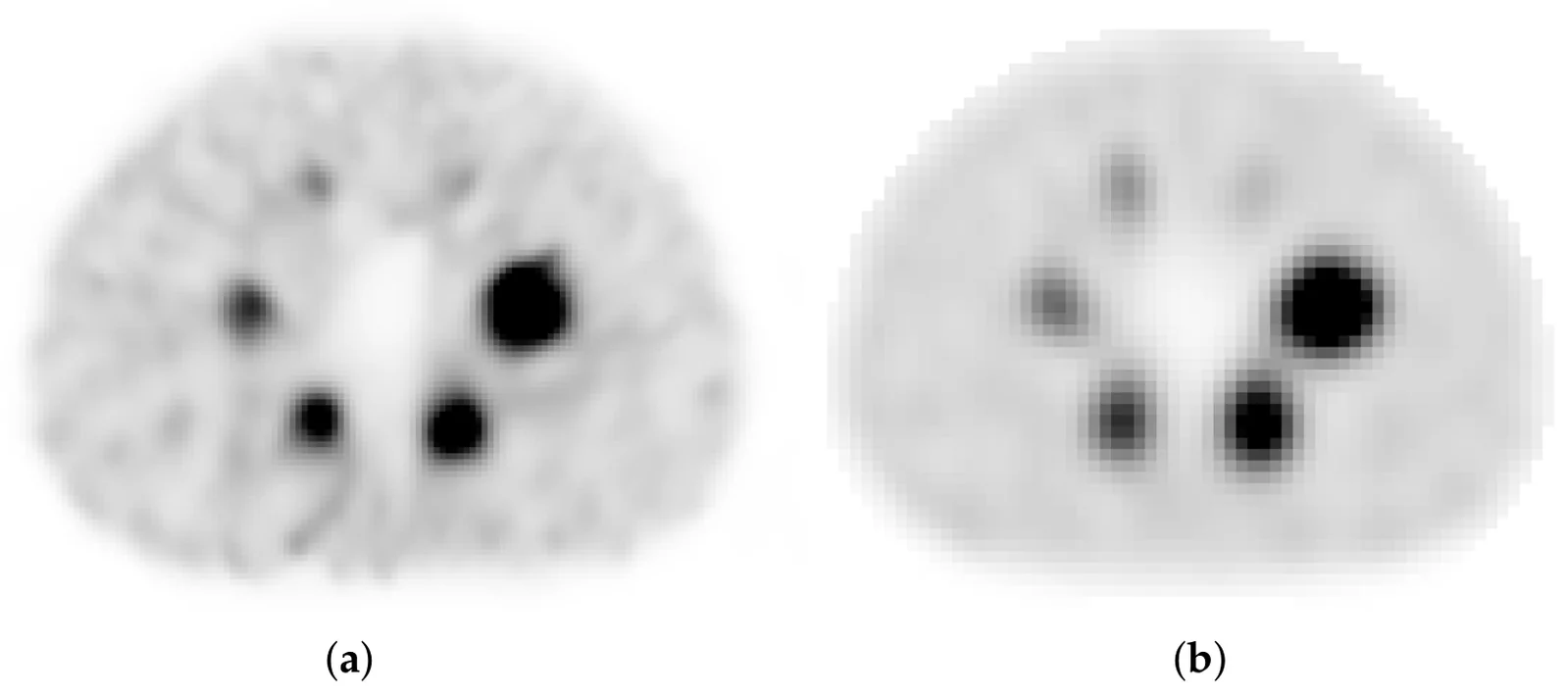
Axial views of the ^177^Lu-filled NEMA phantom reconstructed using the selected candidate optimised StarGuide/Q.Clear settings within the investigated conditions and the Symbia/OSEM settings: (**a**) Protocol ^177^*Lu*_*R*6. (**b**) Protocol ^177^*Lu*_*Symbia*.

These findings are consistent with Figure 8, where clear differences in texture are observed between the StarGuide/Q.Clear reconstruction—noisier and grainier—and the Symbia/OSEM reconstruction—more uniform—when comparing the phantom’s background.

**Figure 9:**
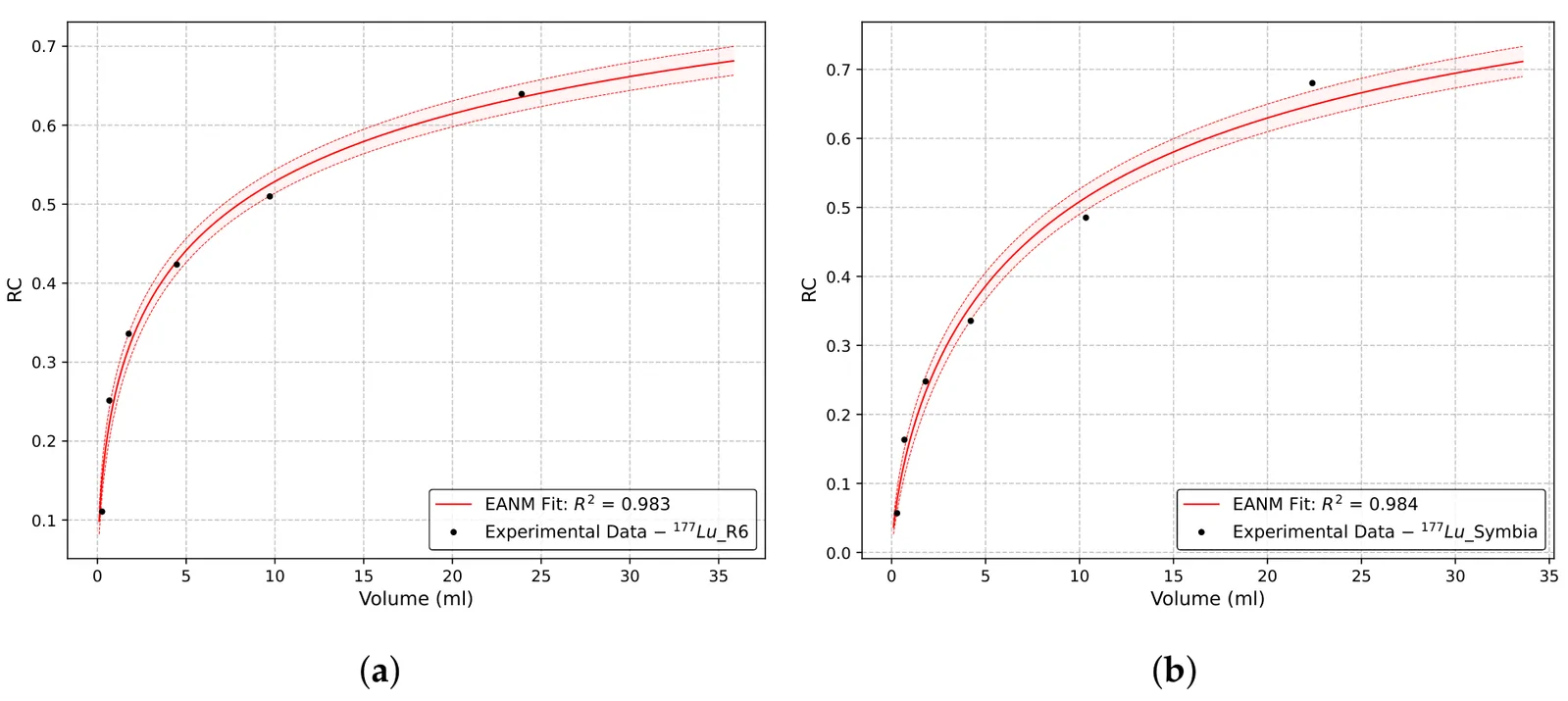
Recovery coefficients and the corresponding EANM fits for ^177^Lu: (**a**) ^177^*Lu*_*R*6 dataset. (**b**) Symbia/OSEM dataset. The dotted red lines represent the lower and upper limits of the 95% confidence band for the fitted function (red solid line).

Both datasets were fitted to the EANM model and were accurately described by it, following the expected trend, as confirmed by the $R^{2}$ values of 0.983 for the StarGuide/Q.Clear reconstruction and 0.984 for the Symbia/OSEM reconstruction (Figure 9).

## 4. Discussion

This study systematically evaluated the influence of the regularisation parameters β and γ on the quantitative performance of StarGuide for ^123^I and optimised the Q.Clear reconstruction parameters for both ^123^I and ^177^Lu. Overall, β produced the largest variations in the evaluated metrics within the studied parameter space, controlling the trade-off between activity recovery and image noise, whereas γ had little measurable influence over the investigated range. Higher β values reduced the RCs, CRCs, and BV while generally increasing the SNR, and the same behaviour was observed for both radionuclides.

These findings are consistent with the stronger image smoothing imposed by a larger penalty factor through RDP regularisation, resulting in greater suppression of high-activity regions. As the VOIs used to calculate the metrics are fixed, the activity recovered within them is reduced, leading to lower RCs and CRCs. Likewise, the results obtained for the BV and SNR reflect the greater noise suppression provided by stronger regularisation. A clear exception was the 0.5mL sphere, for which SNR decreased at higher β values, due to its smaller size causing it to become increasingly blended into the phantom’s background as the smoothing effect overpowered signal recovery.

Although optimisation of the β parameter has only recently been explored in the SPECT domain [5,15,16], it has been extensively studied for Q.Clear in PET systems. Despite the differences between the two imaging modalities, the reductions in the RCs and CRCs with increasing β are in agreement with the findings of Lysvik et al. [34] and, for CRCs, Tian et al. [35], suggesting that the influence of this parameter may be consistent across imaging modalities.

The candidate optimised StarGuide/Q.Clear protocols represented a compromise between quantitative accuracy and image noise, although further optimisation is still required. While StarGuide/Q.Clear generally achieved higher RCs and CRCs than Symbia/OSEM, it underperformed in terms of the BV and SNR. The higher RCs obtained with the Q.Clear reconstructions differ from the results reported by Danieli et al. [8], in which GE-OSEM (GE Healthcare’s implementation of OSEM available on StarGuide) yielded higher RCs than Q.Clear with RDP regularisation. Nevertheless, it is important to note that the OSEM algorithm used in the present study was that of the Symbia Pro.specta, and therefore no strictly direct comparison can be made.

Although the origin of the unexpected reduction in RCs observed for the ^123^I Q.Clear protocols from the 1.2mL sphere to the 2.6mL sphere remains unclear, several factors may have contributed to this phenomenon. The high-energy emissions of ^123^I may have resulted in increased septal penetration and associated scatter, thereby compromising the quantitative accuracy of the images, particularly for the smaller NEMA spheres. Additionally, for both radionuclides, a sharp peak in SNR was observed for the 1.2mL sphere, suggesting that this behaviour may be related either to the calculation methodology employed, including VOI positioning and possible voxelisation effects, or to the behaviour of Q.Clear itself for objects of this size, rather than to a radionuclide-specific phenomenon. As the calibration factors (*CF*s) were applied to all images by multiplying each voxel value, expressed in counts, by 1/CF (Section S1 of the Supplementary Material), an error in the calibration factor would be expected to introduce a systematic scaling effect across all spheres within a given protocol, rather than a sphere-specific effect. Consequently, given the localised nature of the observed discrepancies, an error in calibration is unlikely to fully explain the observed effects. Further investigation, particularly by varying the position of the clone VOIs, should be considered to assess whether systematic errors related to VOI positioning may have contributed to the observed behaviours.

Regarding ^177^Lu quantitative imaging, recent work has evaluated upgraded StarGuide scatter-compensation methods, including modified energy windows and correction for low-energy tail photons [36], relative to the original DEW and TEW methods used in this study. While performance at 208 keV was largely unchanged, the newer methods increased the lesion activity–concentration bias at 113 keV despite improved precision, while improving total-activity estimation [36]. Their integration could therefore affect the 113 keV-derived metrics and potentially the averaged image, but this was not assessed and should be investigated in future work.

Overall, these findings suggest that Q.Clear can play a prominent role in applications requiring accurate quantification, such as personalised dosimetry and theranostics. Particularly, the improved settings identified for ^123^I and ^177^Lu may help improve lesion detectability, although further clinical validation is needed to assess this potential benefit. In contrast, visual interpretation may benefit from stronger regularisation or the use of OSEM. Consequently, reconstruction settings should be selected according to the intended clinical application.

One limitation of this study is the lack of a systematic evaluation of parameters such as the number of iterations, the number of subsets, and the use of tools such as By Sens, without studying their impact on and interaction with other parameters. Additionally, this study used a fixed and well-established geometry, namely, the NEMA phantom, and further investigation is warranted to evaluate the influence of the regularisation parameters when applied to other phantom geometries [8] and to assess their clinical impact using anthropomorphic phantoms and/or patient datasets. Such studies would help determine whether the observed improvements in quantitative phantom performance are clinically relevant, with potential implications for dosimetry and SUV estimation, as well as for qualitative assessment of patient image quality. Another limitation of this study is the lack of a blinded observer assessment of image quality and comparison between the two systems. Future work should incorporate blinded visual assessment to further evaluate the qualitative performance of the reconstructed images. Additionally, the selection of the suggested parameters and protocols relied on a qualitative assessment of the behaviour of the evaluated image-quality metrics and a visual assessment of the NEMA phantom images. Future work would benefit from quantitative, statistically based selection criteria to improve the objectivity and reproducibility of the parameter-selection process. Furthermore, repeatability assessments, such as repeated phantom acquisitions, should be considered in future investigations to enable a robust uncertainty analysis, which was not performed in the present study.

Overall, this study provides a systematic characterisation of the influence of the Q.Clear regularisation parameters on quantitative StarGuide reconstruction. Given the complexity of Q.Clear, standardising its implementation across clinical and research centres remains challenging. Future work should therefore attempt to systematically evaluate reconstruction parameters starting from a basic reconstruction configuration rather than relying on existing clinical protocols or literature-reported reconstruction settings. The systematic evaluation of individual reconstruction settings facilitates the development of standardised quantitative reconstruction protocols for StarGuide and clinically relevant radionuclides, complementing previous optimisation and harmonisation studies, such as those conducted by Cecchin et al. [16].

## 5. Conclusions

This study provides a systematic evaluation of the Q.Clear regularisation parameters for quantitative ^123^I StarGuide SPECT imaging, suggesting that the appropriate selection of β and γ governs the trade-off between quantitative accuracy and image noise. Additionally, candidate optimised Q.Clear reconstruction settings for both ^123^I and ^177^Lu were identified within the evaluated parameter space, providing a reference for future quantitative imaging studies. Nevertheless, the candidate optimised settings are dependent on the system, acquisition protocol, radionuclide, phantom, and image analysis software. Overall, these findings establish a foundation for further Q.Clear protocol optimisation and represent a step towards the standardisation of quantitative StarGuide SPECT reconstruction.

## Figures and Tables

**Table 1:** Acquisition parameters used for the StarGuide and Symbia Pro.specta systems.

Parameter	StarGuide	Symbia Pro.specta
^123^I		
Collimator	LEHR	LEHR
Main energy window	159±10%keV	159±15%keV
Scatter window(s)	131±9%keV	123.12±15%keV 182.8±15%keV
SPECT image matrix	124×124	128×128
SPECT voxel size	$2.46\times 2.46\times 2.46\;{\mathrm{mm}}^{3}$	$2.46\times 2.46\times 2.46\;{\mathrm{mm}}^{3}$
Tube voltage	80kV	110kV
Tube current	70mA	13mA
CT image matrix	512×512	512×512
CT voxel size	$1.0\times 1.0\times 2.5\;{\mathrm{mm}}^{3}$	$0.6\times 0.6\times 3.0\;{\mathrm{mm}}^{3}$
^177^Lu		
Collimator	LEHR	MELP
Main energy window	113±10%keV 208±6%keV	208±20%keV
Scatter window(s)	113 keV: 93.5±11%keV, 133±8%keV 208 keV: 182.7±10%keV	177.1±10%keV 239.6±10%keV
SPECT image matrix	1st. acquisition: 256×256, 2nd. acquisition: 268×268	128×128
SPECT voxel size	$2.46\times 2.46\times 2.46\;{\mathrm{mm}}^{3}$	$2.46\times 2.46\times 2.46\;{\mathrm{mm}}^{3}$
Tube voltage	120kV	110kV
Tube current	60mA	60mA
CT image matrix	512×512	512×512
CT voxel size	$1.0\times 1.0\times 2.5\;{\mathrm{mm}}^{3}$	$1.2\times 1.2\times 3.0\;{\mathrm{mm}}^{3}$

**Table 2:** Combinations of β and γ used for the reconstruction of the ^123^I data.

	β = 0.01	β = 0.05	β = 0.1	β = 0.5	β = 1
γ=0.1	^123^*I*_*R*1	^123^*I*_*R*2	^123^*I*_*R*3	^123^*I*_*R*4	^123^*I*_*R*5
γ=0.5	^123^*I*_*R*6	^123^*I*_*R*7	^123^*I*_*R*8	^123^*I*_*R*9	^123^*I*_*R*10
γ=1	^123^*I*_*R*11	^123^*I*_*R*12	^123^*I*_*R*13	^123^*I*_*R*14	^123^*I*_*R*15
γ=3	^123^*I*_*R*16	^123^*I*_*R*17	^123^*I*_*R*18	^123^*I*_*R*19	^123^*I*_*R*20

**Table 3:** StarGuide/Q.Clear reconstruction protocols applied to the ^177^Lu data.

Protocol	Peak	Iterations	Subsets	γ	β	C3D	By SENS
^177^*Lu*_*R*1	113 keV	12	16	1	0.005	No	OFF
	208 keV	12	16	1	0.005	No	OFF
^177^*Lu*_*R*2	113 keV	20	10	1	0.08	No	ON
	208 keV	20	10	1	0.08	No	ON
^177^*Lu*_*R*3	113 keV	30	10	1	0.3	0.01	OFF
	208 keV	20	10	1	0.008	0.01	OFF
^177^*Lu*_*R*4	113 keV	8	10	1	0.3	0.01	ON
	208 keV	8	10	1	0.02	0.01	ON
^177^*Lu*_*R*5	113 keV	20	10	1	0.08	No	ON
	208 keV	8	10	1	0.02	0.01	ON
^177^*Lu*_*R*6	113 keV	20	10	1	0.2	0.01	ON
	208 keV	8	10	1	0.02	0.01	ON
^177^*Lu*_*R*7	113 keV	20	10	1	0.3	0.01	ON
	208 keV	8	10	1	0.02	0.01	ON
^177^*Lu*_*R*8	113 keV	10	10	1	0.08	0.01	ON
	208 keV	8	10	1	0.02	0.01	ON
^177^*Lu*_*R*9	113 keV	20	10	1	0.08	0.01	ON
	208 keV	8	10	1	0.02	0.01	ON

## Data Availability

The original data presented in the study are openly available at https://doi.org/10.5281/zenodo.22102552. The raw CT and SPECT data supporting the conclusions of this article will be made available by the authors upon request.

## Supplementary Materials

The following supporting information can be downloaded at https://www.mdpi.com/article/10.3390/bioengineering13091004/s1, Section S1: Workflow Development, Calibration and Quantification; Figure S1: Templates developed for the modified MIM workflow: (**a**) NEMA sphere template. (**b**) Background spheres. (**c**) Clone spheres. (**d**) Final positioning of all VOIs; Figure S2: Axial views of the ^123^I-filled NEMA phantom reconstructed with different γ and β combinations; Figure S3: Results of the systematic evaluation of γ: (**a**) Recovery coefficients as a function of γ. (**b**) Background variability as a function of γ. (**c**) Signal-to-noise ratio γ. (**d**) Contrast recovery coefficients as a function of γ; Figure S4: Recovery coefficients for the NEMA spheres for reconstruction protocols ^177^*Lu*_*R*1 to ^177^*Lu*_*R*4; Figure S5: Recovery coefficients for the NEMA spheres obtained for peak and averaged images using the following reconstruction protocols: (**a**) Protocol ^177^*Lu*_*R*2. (**b**) Protocol ^177^*Lu*_*R*4; Figure S6: Comparison of the quantitative image-quality metrics obtained for the 113keV, 208keV and averaged images for protocol ^177^*Lu*_*R*6: (**a**) Recovery coefficients. (**b**) Background variability. (**c**) Signal-to-noise ratio. (**d**) Contrast recovery coefficients; Figure S7: Axial views of the NEMA phantom for the 113keV, 208keV and averaged images for protocol ^177^*Lu*_*R*6: (**a**) 113keV image. (**b**) 208keV image. (**c**) Averaged image.

## Abbreviations

The following abbreviations are used in this manuscript:

BSREM	Block sequential regularised expectation maximisation
BV	Background variability
CF	Calibration factor
CRC	Contrast recovery coefficient
CT	Computed tomography
CZT	Cadmium zinc telluride
DEW	Dual energy window
EANM	European Association of Nuclear Medicine
FWHM	Full width at half maximum
MELP	Medium-energy low-penetration
MIBG	Metaiodobenzylguanidine
MRP	Median root prior
OSEM	Ordered subsets expectation maximisation
PL	Penalised likelihood
RC	Recovery coefficient
RDP	Relative difference penalty
SNR	Signal-to-noise ratio
SPECT	Single-photon emission computed tomography
SUV	Standardised uptake value
TEW	Triple energy window
TRT	Targeted radionuclide therapy
