# Supplementary material for: Optimisation of Q.Clear (BSREM) Reconstruction Parameters for 123I and 177Lu Imaging with a 360° CZT Detector
Source: Bioengineering (Basel). 2026 Aug 28;13(9):1004. doi: 10.3390/bioengineering13091004 (PMC13603108; doi:10.3390/bioengineering13091004)
Supplement: Supplementary file 1 [file bioengineering-13-01004-s001.zip › bioengineering-4500816-supplementary.pdf]

Supplementary Material

Section S1: Workflow Development, Calibration and Quantification

The *QC - NEMA Phantom* workflow, available on MIM Software, uses a VOI template of the six spheres of the NEMA phantom to record count data for each sphere. This workflow was modified to enable conversion from counts to activity concentration, allowing statistical data to be presented and stored without the need for a separate tool, by integrating the determination of the calibration factor and the unit conversion within the original workflow. To achieve this, two new VOI templates were created to replace the original one: the NEMA spheres template and the background spheres template.

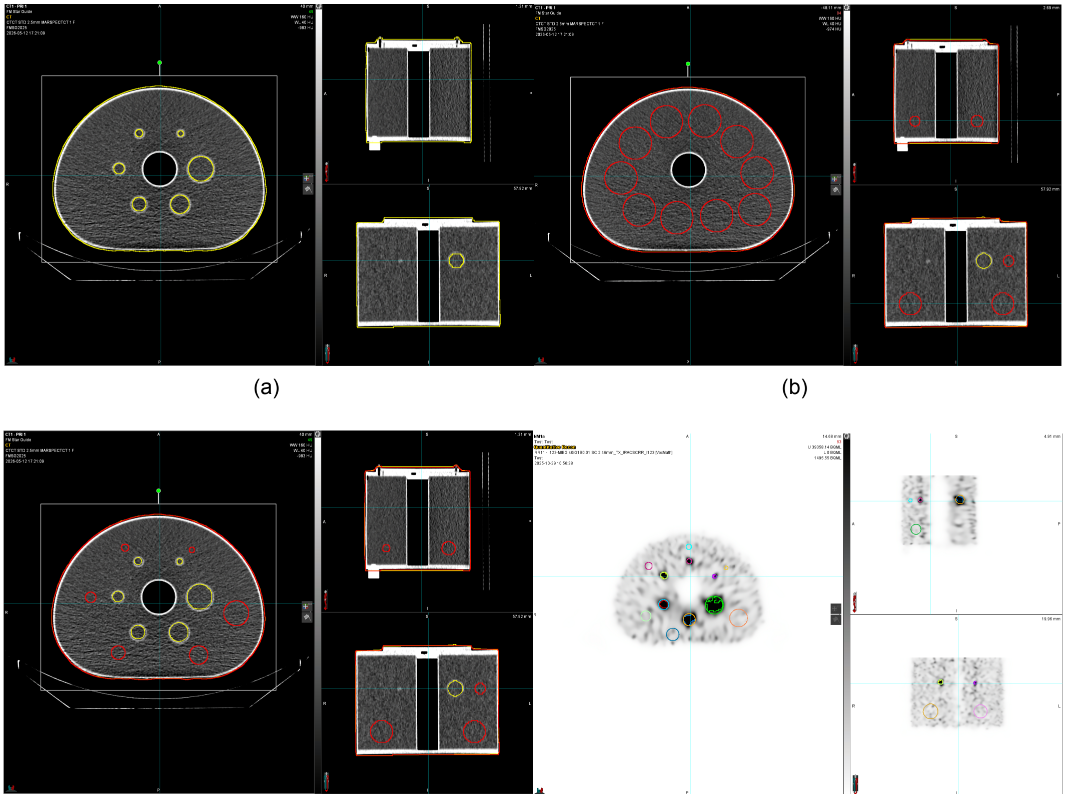

(a) (b) (c) (d)

Figure S1: Templates developed for the modified MIM workflow: **(a)** NEMA sphere template. **(b)** Background spheres. **(c)** Clone spheres. **(d)** Final positioning of all VOIs on the SPECT scan following template confirmation and completion of the required user inputs.

The NEMA spheres template consists of a contour of the main cylinder, which facilitates template positioning, and six spheres matching the spherical inserts (Figure S1(a)). These spheres were drawn to match the internal diameters of the NEMA spheres using the CT axial slice where they appeared to be the widest and their positioning was further verified using the coronal and sagittal views. The background template consists of a contour of the main cylinder, ten spheres with a volume of $51.0\,ml$, and six additional spheres identical to the NEMA spheres. The ten spheres were evenly distributed within the phantoms' background, using the CT scan for guidance, away from both the spherical inserts and the phantom walls (Figure S1(b)). The remaining six spheres were placed adjacently to each corresponding NEMA sphere, in the same plane used

to define them (Figure S1(c)). For simplicity the ten spheres are hereafter referred to as the background spheres, while the remaining spheres in the background template are referred to as the clone spheres. The clone spheres were used to estimate background contributions within an equivalent volume for each NEMA sphere.

To convert image counts to activity concentration, MIM's *Z-launch convert to Bq/ml* unit conversion algorithm was integrated in the workflow. The conversion is given by equation:

$$AC = \frac{C}{T \times V \times CF}\,, \tag{1}$$

where C are the image counts, AC is the activity concentration, T is the acquisition time, V is the reconstructed voxel volume, and CF is the calibration factor. V is retrieved from the DICOM header and for the Symbia Pro.specta, T is calculated from the following equation

$$T = N_{FR} \times T_F \times N_D\,, \tag{2}$$

where N_{FR} is the number of frames in rotation, T_F is the actual frame duration and N_D is the number of detectors, all retrieved from the DICOM header. Equation 2 led to incorrect estimations of acquisition when used on the StarGuide system. Therefore, the acquisition time for this system is provided as user input. CF is calculated using the following equation

$$CF = \frac{\overline{C}_{BG}}{\overline{V}_{BG} \times c_{BG} \times T}\,, \tag{3}$$

where $\overline{C}_{BG}$ is the average total counts in the background spheres, c_{BF} is the background activity concentration and $\overline{V}_{BG}$ is the average volume of the background spheres.
Due to the difference in resolution between the CT and SPECT scans, MIM Software automatically adapts the spheres diameter to better match the SPECT images, sightly changing their volume in the process. Consequently, the average volume of the ten background spheres was used to calculate the calibration factor. The background activity concentration was implemented as a user input for both SPECT/CT systems, in the same manner as the acquisition time for the StarGuide system.
For Symbia Pro.specta, an additional step was included in the developed workflows. The system automatically retrieves the pixel size factor from the DICOM header and divides the image counts by this factor. In contrast to the StarGuide system, where the pixel size factor is defined by the user prior to acquisition, and was set to 1 for all acquisitions considered in this study, the Symbia Pro.specta system automatically defines this factor to compensate, for example, for low statistics, making this correction necessary.
After template placement and completion of all user inputs, the workflow automatically creates a CSV file containing the following information: total activity within each VOI (Bq), maximum and mean activity concentration within each VOI (Bq/ml), the standard deviation of the mean activity concentration within each VOI (Bq/ml), and the volume of each VOI (ml).

Figures

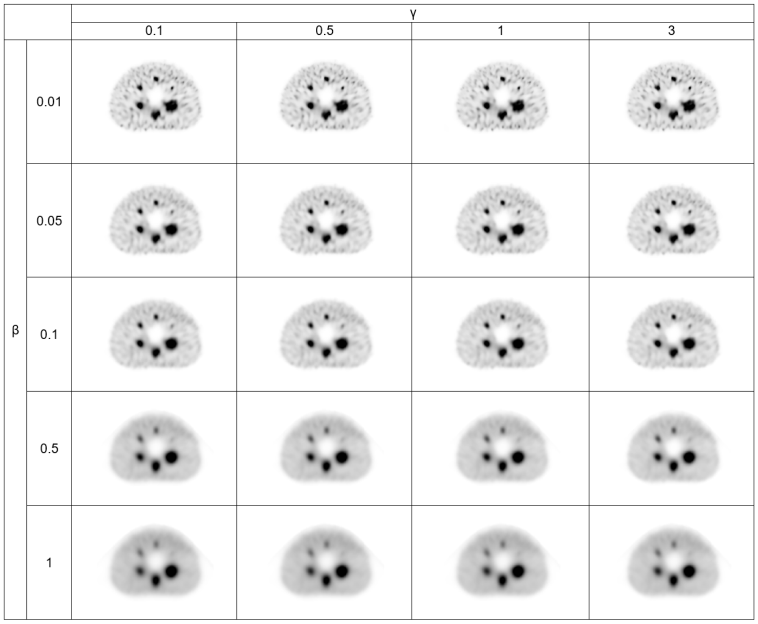

Figure S2: Axial views of the ^{123}-filled NEMA phantom reconstructed with different γ and β combinations.

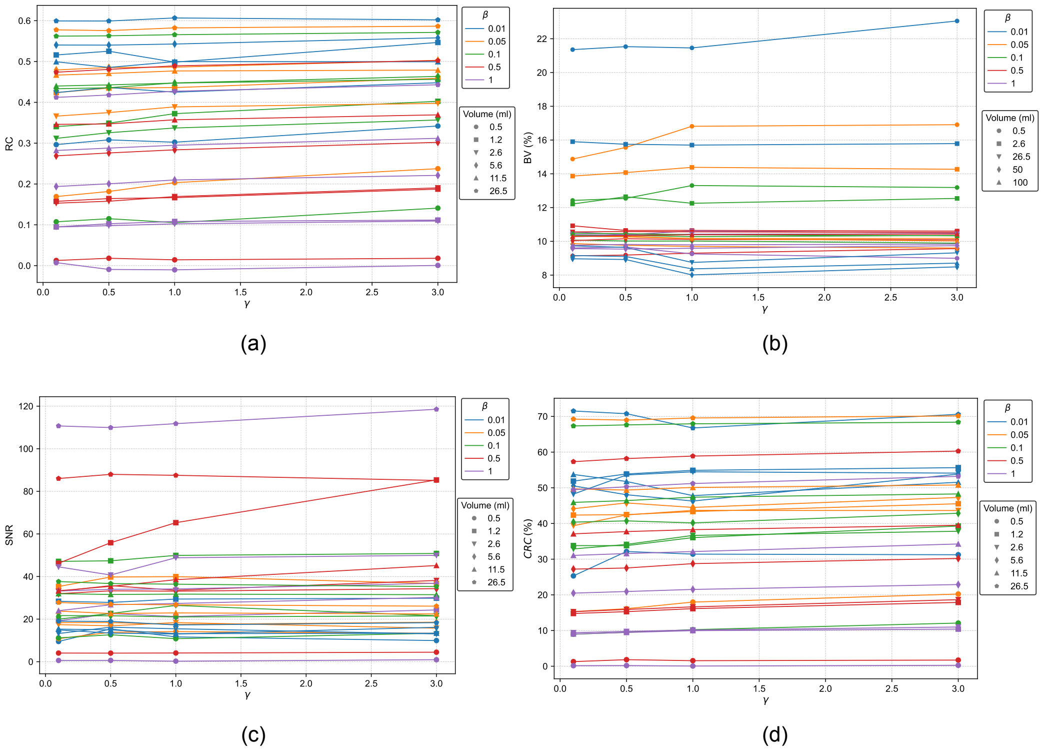

Figure S3: Results of the systematic evaluation of γ: **(a)** Recovery coefficients as a function of γ. **(b)** Background variability as a function of γ. **(c)** Signal-to-noise ratio γ. **(d)** Contrast recovery coefficients as a function of γ.

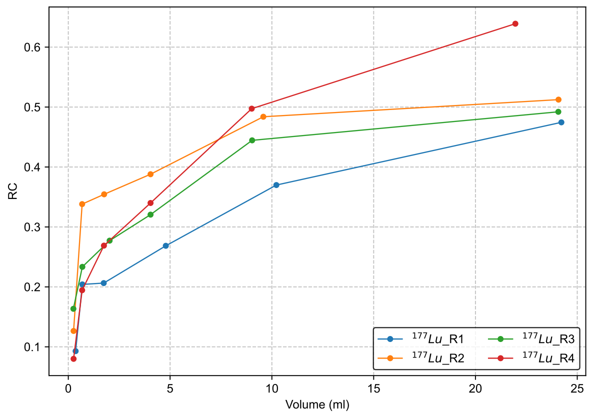

Figure S4: Recovery coefficients for the NEMA spheres for reconstruction protocols $^{177}Lu\_R1$ to $^{177}Lu\_R4$.

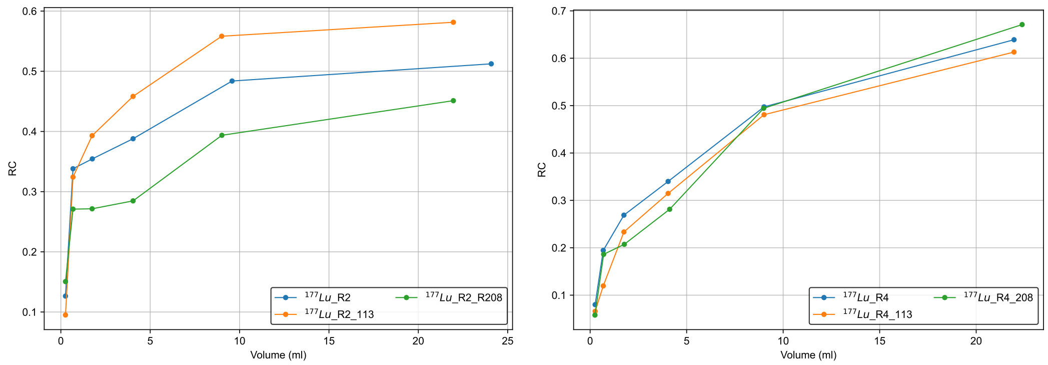

(a) (b)

Figure S5: Recovery coefficients for the NEMA spheres obtained for peak and average images using the following reconstruction protocols: **(a)** Protocol $^{177}Lu\_R2$. **(b)** Protocol $^{177}Lu\_R4$.

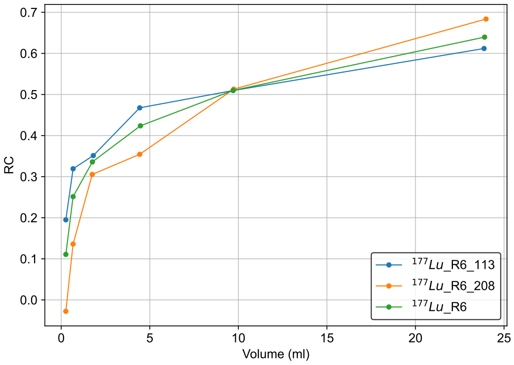

(a)

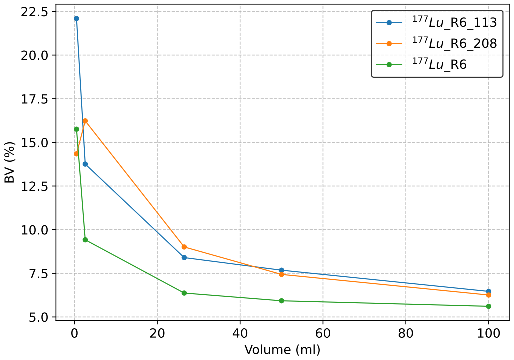

(b)

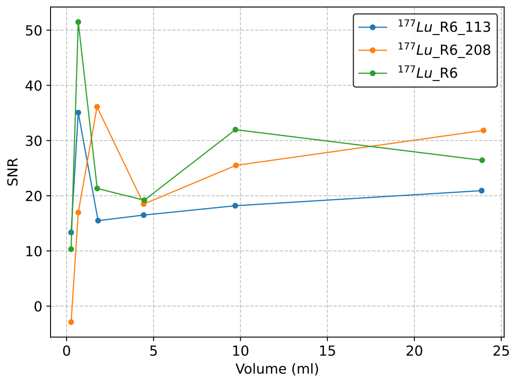

(c)

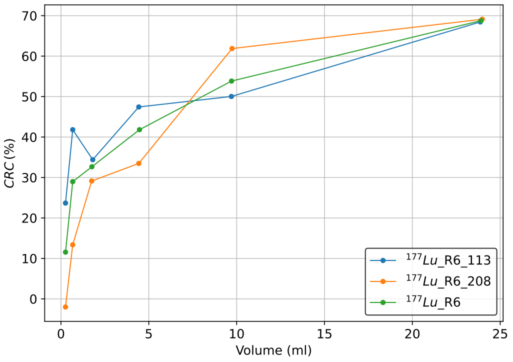

(d)

Figure S6: Comparison of the quantitative image quality metrics obtained for the 113 keV, 208 keV and averaged images for the protocol $^{177}Lu\_R6$: **(a)** Recovery coefficients. **(b)** Background variability. **(c)** Signal-to-noise ratio. **(d)** Contrast recovery coefficients.

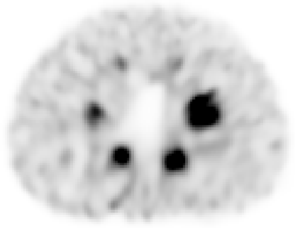

(a)

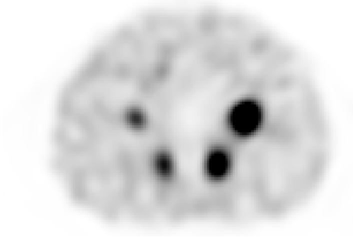

(b)

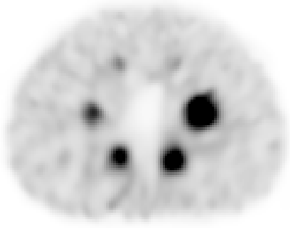

(c)

Figure S7: Figure S7: Axial views of the NEMA phantom for the 113 keV, 208 keV and averaged images for protocol $^{177}Lu\_R6$: **(a)** 113 keV image. **(b)** 208 keV image. **(c)** Averaged image.
